# Do *Ganoderma* Species Represent Novel Sources of Phenolic Based Antimicrobial Agents?

**DOI:** 10.3390/molecules28073264

**Published:** 2023-04-06

**Authors:** Milena Rašeta, Jovana Mišković, Eleonora Čapelja, Ewa Zapora, Aleksandra Petrović Fabijan, Petar Knežević, Maja Karaman

**Affiliations:** 1 Department of Chemistry, Biochemistry and Environmental Protection, Faculty of Sciences, University of Novi Sad, Trg Dositeja Obradovića 3, 21000 Novi Sad, Serbia; 2 Department of Biology and Ecology, Faculty of Sciences, University of Novi Sad, Trg Dositeja Obradovića 2, 21000 Novi Sad, Serbia; 3 Institute of Forest Sciences, Białystok University of Technology, Wiejska 45E, 15-351 Białystok, Poland; 4 Centre for Infectious Diseases and Microbiology, Westmead Institute for Medical Research, 176 Hawkesbury Road, Westmead, NSW 2145, Australia

**Keywords:** *G. applanatum*, *G. lucidum*, *G. pfeifferi*, *G. resinaceum*, antibacterial activity, antifungal activity, antiviral activity, extracts, phenolics

## Abstract

*Ganoderma* species have been recognized as potential antimicrobial (AM) agents and have been used in traditional Chinese medicine (TCM) for a long time. The aim of this study is to examine the AM potential of autochthonous *Ganoderma* species (*G. applanatum*, *G. lucidum*, *G. pfeifferi* and *G. resinaceum*) from Serbia. The extraction of fungal material was prepared in different solvents (ethanol—EtOH, water—H_2_O, chloroform—CHCl_3_). Antibacterial activity (ABA) was determined using disk-diffusion, agar-well diffusion, and micro-dilution method, while for antifungal properties disk-diffusion and pour plate method were applied. Antiviral activity was tested on model DNA virus LK3 and determined by plaque assay. Statistical PCA analysis was applied for detection of correlation effects of phenolics and AM activities, while LC-MS/MS was performed for phenolics quantification. *G. resinaceum* CHCl_3_ extract expressed the most potent ABA against *P. aeruginosa* (MIC = 6.25 mg/mL), probably due to presence of flavonoids and 2,5-dihydroxybenzoic acid. Among H_2_O extracts, the highest ABA was determined for *G. pfeifferi* against both *E. coli* and *S. aureus* (21 and 19 mm, respectively). EtOH extracts of *G. pfeifferi* and *G. resinaceum* were the most effective against *A. niger* (23.8 and 20.15 mm, respectively), with special impact of phenolic acids and flavonoid isorhamnetin, while *C. albicans* showed the lowest susceptibility. The most potent antiviral inhibitor was *G. lucidum* (70.73% growth inhibition) due to the high amount of phenolic acids. To the best of our knowledge, this is the first report of a methodical AM profile of *G. pfeifferi* and *G. resinaceum* from the Balkan region including PCA analysis.

## 1. Introduction

One of the global public health threats to humans is the emergence of multi-drug-resistant organisms, which compromise the capacity of medical professionals to treat an expanding range of infectious diseases [1,2]. This follows increased drug-dose consumption, longer hospital stays, higher toxicity of drugs, and increased mortality [3]. One of the most common micro-organisms that have developed multi-resistance are *Staphylococcus aureus, Enterococcus faecium*, *Enterococcus faecalis* [4], *Streptococcus pneumonia*, *Acinetobacter baumannii, Mycobacterium tuberculosis, Pseudomonas aeruginosa, Salmonella enterica* and *Vibrio cholerae* [5]. Consequently, the creation of novel, more effective pharmaceuticals to combat current antibiotic-resistant bacteria is urgently needed.

Nonetheless, systemic fungal infections are on the rise, especially in immunocompromised and post-operative patients [6]. Moreover, mucormycosis is becoming a serious issue for COVID-19 patients in intensive care units who are susceptible to opportunistic fungal pathogens. Since current available treatments are less promising and less effective, researchers are focusing on finding new agents against mucormycosis and other fungal infections [7]. Furthermore, the increased use of antifungal agents is resulting in the development of resistance to present drugs.

Genus *Ganoderma* is one of the most widely distributed genera of fungi with the majority of species found in tropical and subtropical regions of Africa, America, Asia, and Oceania, though a few species can also be found in Europe [8]. Despite wide distribution, just a few species, mainly from *G. lucidum* complex (currently thirteen well-delineated species recognized in this group, including *G. resinaceum*), were used in traditional Chinese Medicine (TCM) for over two millennia and over the last few decades it has gained a lot of interest in Western medicine [9]. In addition to its well-studied anticancer, antioxidative, hepatoprotective and immunomodulatory activities [10], various *Ganoderma* extracts have also been found to have antibacterial (AB) activity [11,12,13,14,15,16,17], as well as antifungal (AF) [18,19] and antiviral (AV) [20,21] properties. The presence of phenolic compounds was linked to the AB activity of extracts from *G. lucidum* [12,22,23] and *G. resinaceum* [24], while terpenoids from *G. applanatum* have been connected to the antioxidant (AO) and antimicrobial (AM) activities [14]. AF activity against some filamentous fungal species, such as *Fusarium*, *Penicillium* and *Aspergillus*, as well as EtOH extracts of both *G. resinaceum* and *G. pfeifferi*, was linked to the presence of the *p*-coumaric and vanillic acid [25]; there is also evidence that phenols, such as carvacrol and thymol, may have better efficiency than phenolic acids [26]. *G. pfeifferi* and *G. lucidum* extracts showed AV activity, while triterpenoids and some volatile compounds were detected as the main constituents responsible for this activity [27,28].

AM activity of phenolic compounds is probably related to the site and number of hydroxyl groups on the phenol group—increased hydroxylation results in increased toxicity towards the target micro-organism. This is manifested through enzyme inhibition by the oxidized compounds, probably in reaction with sulfhydryl groups or through more nonspecific interactions with the proteins [29]. In fungal cells, toxicity of phenolics is attributed to the binding of these compounds to ergosterol in cell membranes as well as to the inhibition of enzymes involved in synthesis of sterols [30]. Considering the AM mechanism of terpenoids, it was determined that monoterpenes induce membrane disruption [31], while diterpenes inhibit oxygen uptake and induce oxidative phosphorylation in bacterial cells [32].

The aim of this study was to quantify selected phenolic compounds and determine AB, AF and AV properties of H_2_O, EtOH and CHCl_3_ extracts of four *Ganoderma* species: *G. applanatum*, *G. lucidum*, *G. pfeifferi* and *G. resinaceum*. PCA statistical analysis of AM profiles (obtained AM activities) in relation to identified phenolic compounds is presented for the first time in this study.

## 2. Results and Discussion

### 2.1. Mycochemical Profile by LC-MS/MS Detection

The results of both chemical analyses (LC–MS/MS) and biological activities (anti-inflammatory, antioxidant, antidiabetic, antiproliferative) of selected *Ganoderma* species (EtOH and H_2_O extracts) have been published in our previous studies [23,25,33] (Table S1). Chemical identifications of *G. applanatum* and *G. lucidum* EtOH and H_2_O extracts were published in 2016 [23], while LC–MS/MS results of the same extracts for *G. pfeifferi* and *G. resinaceum* were published in 2020 [25]. A new mycochemical profile of all mentioned species was reported in 2020 [33]. On the other hand, in the present study, quantifications of phenolic compounds of CHCl_3_ extracts of *G. applanatum* and *G. resinaceum* were conducted using LC-MS/MS analysis, due to examination of AB activity and identification of potentially active secondary metabolites (Table 1).

Phenolic acids (*p*-hydroxybenzoic acid, protocatechuic acid, *p*-coumaric acid, vanillic acid, gallic acid, caffeic acid, quinic acid, syringic acid, chlorogenic acid), coumarin (aesculetin) and flavonoids (chrysoeriol and isorhamnetin) were identified in *Ganoderma* species and described in our previous studies [23,25,33]. In general, EtOH extracts contained higher amounts of phenolic compounds, where *p*-hydroxybenzoic acid (23 μg/g d.w.), gallic acid (30.50 μg/g d.w.), vanillic acid (11.40–12.10 μg/g d.w.), protocatechuic acid (22.40–23.20 μg/g d.w.), quinic acid (10.90 μg/g d.w.) and syringic acid (9.80 μg/g d.w.) stood out [23,25,33]. Among H_2_O extracts, the *p*-hydroxybenzoic, protocatechuic, vanillic and quinic acid were the most abundant compounds [23,25,33]. Results are in accordance with identification analysis data of polar extracts (MeOH and H_2_O) of *G. applanatum* and *G. resinaceum* from Turkey, where nine phenolic compounds were found, with the highest level of apigenin in MeOH extract of *G. resinaceum* (1761 ± 15 μg/g extract) while (+)-catechin was present in both species, ranging from 76 to 287 μg/g extract [24].

In this study, in CHCl_3_ extracts of *G. applanatum* and *G. resinaceum*, six phenolic compounds were detected in highest amounts, including 2,5-dihydroxybenzoic acid (60.12 and 79.50 μg/g d.w., respectively) as well as two flavonoids: apigenin (35.12 and 47.87 μg/g d.w., respectively), rutin (26.20 and 32.18 ug/g d.w., respectively) and amentoflavone (4.13 and 18.95 μg/g d.w., respectively) (Table 1).

The scientific data indicate that chemical screening of CHCl_3_ extracts of *G. applanatum* confirmed the presence of flavonoids and phenols, together with saponins and glycosides [34], while alkaloids, flavonoids, terpenoids and saponins were found in EtOH and H_2_O extracts of *G. lucidum* [1,16]. In contrast to *G. applanatum* and *G. lucidum*, from which more terpenoid compounds and polysaccharides have been isolated, *G. pfeifferi* has received much less attention from mycochemical studies [35].

### 2.2. Antimicrobial Activity

*Ganoderma* species may exhibit various AM activities based on the polarity of the chemicals produced by the extraction solvent. It was previously summarized by Cowan and Plant [29] that different chemical substances can affect a diverse mechanism of AM activity; caffeic acid is one of the substances within a large group of phenylpropane-derived compounds with the greatest oxidation state. Moreover, there is evidence that increasing hydroxylation causes increased toxicity, and that the site(s) and amount of hydroxyl groups on the phenol group are thought to be related to their relative toxicity to microorganisms [29]. Hence, in this study three different solvents were used for detection of AM activity.

#### 2.2.1. Antibacterial Activity

Based on antibiogram results (Table S2), it can be concluded that the largest number of tested strains are sensitive to amikacin, except for *E. faecalis* ^ATCC 19433^, which is resistant. Resistance to tetracycline was shown by: *P. aeruginosa* ^ATCC 35554^, *E. coli* ^ATCC 11229^, whereas the strains *K. aerogenes* ^ATCC 13048^, *B. cereus* ^ATCC 11778^ and *E. coli* ^ATCC 11775^ were of intermediate sensitivity. All bacterial strains showed resistance to methicillin and kanamycin, except for two strains *S. aureus* ^ATCC 6538^ and *S. aureus* ^ATCC 25923^. Additionally, both *B. cereus* ^ATCC 11778^ and *P. aeruginosa* ^ATCC 35554^ were resistant to ceftriaxone, while the other strains proved to be sensitive to this antibiotic. Multi-resistance is observed in *E. coli* ^ATCC 11229^, *B. cereus* ^ATCC 11778^ and *P. aeruginosa* ^ATCC 35554^.

The highest AB activity of H_2_O extracts, using diffusion assays, was observed for *G. pfeifferi* against *E. coli* ^ATCC 11775^ and *B. cereus* ^ATCC 11778^, with inhibition zones of 21 and 19 mm, respectively (Table 2). High susceptibility against these two bacterial strains was also observed for *G. lucidum* and *G. resinaceum* (16 and 13 mm, respectively), while *G. applanatum* showed the highest AB against *S. aureus* ^ATCC 255923^. Diffusion-based approaches against *P. mirabilis* ^ATCC 12453^ yielded no results, while against *P. aeruginosa* only *G. resinaceum* extract was active.

Analyses using three different methods showed that H_2_O extracts had the highest activity against Gram-positive bacterial strains, with some minor activity against Gram-negative bacteria. In addition, MIC and MBC against bacterial strains were determined in an in vitro microdilution test (Table 2), with lower values indicating greater efficacy of the tested extract, i.e., higher AB activity. The following order of activity was obtained: *G. resinaceum* (100 mg/mL) > *G. applanatum* (100 mg/mL) > *G. pfeifferi* (200 mg/mL) > *G. lucidum* (200 mg/mL). The highest MBC activity was demonstrated by *G. resinaceum* on multi-resistant *B. cereus* strain, which confirmed the previous conclusion that H_2_O extracts are more effective against Gram-positive bacteria.

All tested CHCl_3_ extracts of *G. resinaceum* and *G. applanatum* exhibited AB activity (Table 3). The analyzed strains showed significant variation in their MIC and MBC values, which varied in the range: MIC = 6.125–25 mg/mL and MBC = 25–50 mg/mL, indicating the strain specificity of the analyzed micro-organisms. *G. resinaceum* exhibited the highest AB activity (MIC = 6.25 mg/mL) against multi-resistant *P. aeruginosa* ^ATCC 3554^, which was more than twice the strength of the activity of streptomycin (MIC = 16 mg/mL). The CHCl_3_ extracts of this fungal species were the least effective against both *K. pneumoniae* ^HP^ and *B. cereus* ^HP^, whereas high activities against *E. coli* ^ATCC 11229^*, S. aureus* ^ATCC 6538^ and *S. enteritidis* ^ATCC 13076^ (MIC = 12.5 mg/mL) were recorded. *G. applanatum* showed the highest AB activity against multi-resistant *E. coli* ^ATCC 11229^ and *B. cereus* ^HP^ with MIC of 12.5 mg/mL. The MBC values of both fungal species (*G. applanatum* and *G. resinaceum*) against *S. aureus* ^ATCC 6538^, *S. enteritidis* ^ATCC 13076^ and *B. cereus* ^HP^ were recorded at the same concentration of 25 mg/mL, while the values against other bacterial strains differed (Table 2).

Comparing all *Ganoderma* species tested in this research, it is evident that *G. resinaceum* expressed the most potent antibacterial activity. Al-Fatimi et al. [11] compared AB activity of selected basidiomycetes from Yemen, where *G. resinaceum* also stood out with the highest activity. Moreover, Al-Fatimi et al. [11] compared the level of AB activity of three different types of extracts (CH_2_Cl_2_, MeOH and H_2_O). Different AB activity related to different solvents used was also observed in their study, with H_2_O being the most active, especially against *P. aeruginosa* and *S. aureus* (20 mm inhibition zone) [11]. The effectiveness of H_2_O extracts of *G. resinaceum* against these two bacteria strains was also confirmed in another study [16]. H_2_O extracts of *G. lucidum* fruiting body from India exhibited weaker AB activity against *E. coli*, with inhibition zone diameters ranging from 7 to 13 mm [15]. On the other hand, contrary to results from our study, *G. lucidum* extracts from India showed activity against *S. aureus* and *P. aeruginosa* [15], while Mousavi et al. [17] reported good AB activity of MeOH extract against Gram-negative bacteria (*E. coli* and *P. aeruginosa*). Furthermore, H_2_O extracts of *G. lucidum* collected in Turkey and Nepal showed activity against *E. faecalis* and *P. aeruginosa* [1,36], which was not consistent with our results. H_2_O extracts of *G. applanatum* from India exhibited similar AB susceptibility against *S. aureus* (inhibition zone 12 mm) and no activity against *P. aeruginosa*, while higher activity was observed against *E. coli* [34]. Different activities obtained by Karaman et al. [13] on different strains of *S. aureus*, demonstrated that the fungal extracts were target-specific at the intraspecies level, which is also in accordance with the results of this study. *G. pfeifferi* exhibited the highest AB susceptibility against *E. coli* (21 mm), which is in accordance with the report [19]; among Gram-negative bacteria, *E. coli* was the most affected by volatile oil isolated from this fungus (15 mm). Moreover, *G. pfeifferi* volatile oil exhibited the largest inhibition zones against Gram-positive bacteria, including *Bacillus subtilis* (20 mm) and *S. aureus* (30 mm) [19], which also supports the results obtained in this study (Table 2).

The inhibitory action of the extracts against particular bacteria strains is directly correlated with the extraction process [37]. The results obtained for the tested CHCl_3_ extracts of both *G. applanatum* and *G. resinaceum* show stronger AB effects at lower concentrations (25 mg/mL) compared to MeOH (50 mg/mL) [12,38]. Zengin et al. [24] reported weak AB activity of H_2_O and MeOH extracts of these *Ganoderma* species against *E. coli* and *P. aeruginosa.* In contrast, Nagaraj et al. [34] outlined good activity of MeOH extracts against *S. aureus*, *E. coli* and *B. subtilis*, followed by H_2_O, petroleum ether and CHCl_3_ extraction.

In this study, H_2_O extract of *G. resinaceum* was effective against *P. aeruginosa*, but the highest AB activity was observed for CHCl_3_ extract of this fungal species. The fact that AB activity was noticed in extracts with different polarities implies that these *Ganoderma* species contain substances with AB action. Two new hydroquinones isolated from *G. pfeifferi* (ganomycin A and ganomycin B) were confirmed to have AB activity by TLC chromatography [27,39]. Two isolated lanostanoid compounds from *G. applanatum*, 5-ergost-7-en-3-ol, ergost-7,22-dien-3-ol and 5,8-epidioxy-5,8-ergost-6,22-dien-3-ol primarily showed activity against Gram-positive bacteria [40]. Of the non-polar compounds, antimicrobial activity is mainly attributed to terpenoid compounds [40]. The exceptions are ganomycins isolated from *G. pfeifferi,* which are phenolic in nature. Moreover, Nagaraj et al. [34] associated the good AB activity of MeOH extracts of *G. applanatum* with higher solubility of detected active compounds (saponins, phenols, steroids, glycosides, terpenoids and flavonoids) in alcohol. High content of phenols in the form of coumarins, flavonoids and tannins were also detected in *G. applanatum* [23,33,38]. Results are consistent with previous reports which demonstrated that the most active components are typically water insoluble and that low polarity organic solvents would provide more active extracts [12,39,41]. Furthermore, the activity profile of a more lipophilic extract solvent (CHCl_3_) exhibited similarities between *G. resinaceum* and *G. applanatum*, suggesting that the solvent might be extracting comparable chemicals from these *Ganoderma* species. This is also in accordance with the results obtained in the research of different *Ganoderma* species from Nigeria, where CHCl_3_:CH_3_COCH_3_ extracts were the most active [39]. Significant AB activity was observed in similar tests of CHCl_3_ extracts of *G. lucidum*, where predominantly non-polar molecules were documented as distinct antibacterial agents [12,29,42]. For example, in several studies rutin was documented as a potent AB agent against *E. coli* and *P. aeruginosa* [43,44], while Arima et al. [45] reported its synergistic effect with other flavonoids against *B. cereus* [46]. Thus, we can assume that non-polar compounds, such as rutin, detected in this study in CHCl_3_ extract may also be responsible for high AB susceptibility of *G. resinaceum*.

By comparing methods applied in examination of AB activity, we can conclude that microdilution method was the most accurate since the obtained data is clearer and objective. In addition to solvents and different methods of testing AB activity, growing conditions also represent very important factors for assessing AB susceptibility. Jorcin et al. [47] investigated the effects of culture conditions on AB activity of *G. resinaceum* and determined that larger-scale cultures in malt extract broth supplemented with 20 g/L glucose and a 15-day incubation should be carried to isolate antibiotic compounds that may be effective against *S. aureus.* Alves et al. [48] also proved the AM activity of phenolic acids. Based on above results, it is believed that fungal extracts could be a source of substances used as a substitute for commercial antibiotics, to which certain micro-organisms are proven to be resistant.

#### 2.2.2. Antifungal Activity

**Figure 1:**
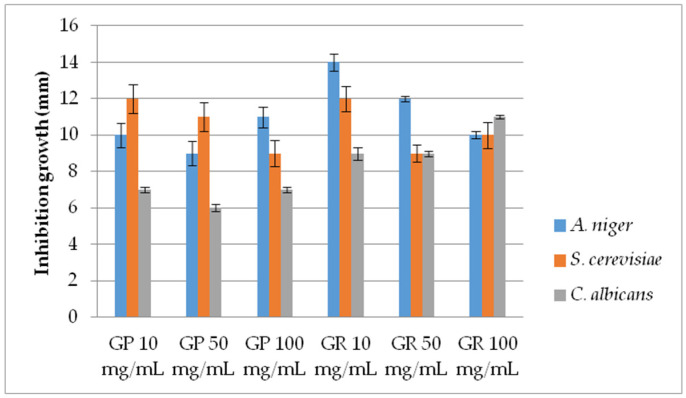
Antifungal activity of *G. pfeifferi* and *G. resinaceum* EtOH extracts using disk-diffusion method. Abbreviations: GP—*G. pfeifferi*; GR—*G. resinaceum*.

**Figure 2:**
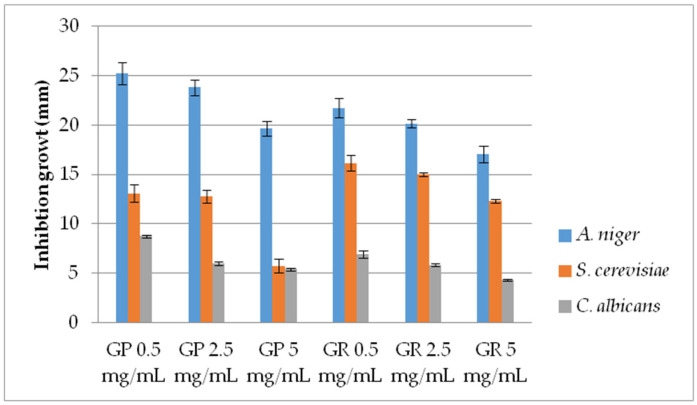
Antifungal activity of *G. pfeifferi* and *G. resinaceum* EtOH extracts using pour plate method. Abbreviations: GP—*G. pfeifferi*; GR—*G. resinaceum*.

AF activity of EtOH extracts (range 10–100 mg/mL) of *G. pfeifferi* and *G. resinaceum* was examined against *S. cerevisiae*, *C. albicans*—yeast and filamentous *Aspergillus niger*, by measuring the inhibition zone (mm) (Figure 1). Moreover, AF of these species was determined by applying the pour plate method for lower extract concentrations (0.5, 2.5 and 5 mg/mL) in order to measure the diameter of mycelia growth in a more precise manner for AF activity determination (Figure 2).

In most cases regarding the disk diffusion method, a concentration dependence was observed, i.e., the level of AF activity increases with the *Ganoderma* extract concentration (Figure 1). However, the highest inhibition was demonstrated by extracts of *G. resinaceum* with the lowest tested conc. (10 mg/mL) against *A. niger*, with an inhibition zone of 14 mm, following *G. pfeifferi* and *G. resinaceum* extracts against *S. cerevisiae* (12 mm) at the same concentration. *G. pfeifferi* was the least effective against *C. albicans* strains, while AF activity of *G. resinaceum* was obtained in the following order: *A. niger* > *S. cerevisiae* > *C. albicans* (Figure 1).

Considering the variations in activity, a more detailed examination of the *Ganoderma* extracts at lower concentrations was conducted by using the pour plate method (Figure 2). AF activity was inversely proportional to the examined concentration of both *Ganoderma* extracts, demonstrating very strong AF capacity, especially against *A. niger*. Furthermore, results are in accordance with growth diameters obtained by the disk-diffusion method since the extracts were most effective against *A. niger*. The lowest activity was shown against *C. albicans* (Figure 1 and Figure 2). *G. pfeifferi* revealed better AF potential against *A. niger* (19.67–25.25 mm) and *C. albicans* (5.42–8.75 mm) compared to *G. resinaceum*, where inhibition zones were in ranges of 17.08 to 21.75 mm and 4.33 to 6.92 mm, respectively.

Obtained results are in accordance with the research of Suansia and John [18], where mycelial growth of both *A. niger* and *A. flavus* was completely inhibited by *Ganoderma* sp. EtOH and MeOH extracts. On the other hand, volatile oil isolated from *G. pfeifferi* was significantly efficient against *C. albicans* (MIC = 0.6 mg/mL) [19], while *C. maltosa* showed resistance to MeOH and H_2_O extracts of *G. resinaceum* [11]. Zengin et al. [24] compared AF activity of *G. applanatum* and *G. resinaceum*, where only *G. applanatum* MeOH extracts showed AF potential against *C. albicans* at a concentration 2.5 mg/mL. Furthermore, *C. albicans* and *C. maltosa* were resistant to sesquiterpenoid hydroquinones and ganomycins A and B from *G. pfeifferi* [27], suggesting that some other metabolites are responsible for AF activity. *p*-Coumaric and vanillic acid identified in EtOH extracts of *G. pfeifferi* and *G. resinaceum* [25,49] were reported as moderate AF agents against *Fusarium oxysporum, F. verticillioides, Penicillium brevicompactum, P. expansum, A. flavus* and *A. fumigatus* with percentage inhibition ranging from 18.70 to 100.00% [26]. Nevertheless, results of a study conducted by Zabka and Pavela [26] showed that phenols had a substantially better efficiency than phenolic acids. Moreover, we can assume that rutin found in this study in the CHCl_3_ extract of *G. pfeifferi* may be responsible for AF activity. Other research also reported this flavonoid as a more potent AF agent against *C. krusei* compared to fluconazole, while moderate action was observed against *C. albicans* [50]. On the other hand, numerous literature data indicate the importance of proteins and polysaccharides, such as glucan [51,52,53,54], for exhibiting AF activity by activating and enhancing the immune response; thus, its use is recommended in combination with other antibiotics and immuno-stimulators in the prevention and treatment of infectious diseases [55]. Wang and Ng [53] isolated ganodermin, a low molecular weight protein (15 kDa) from *G. lucidum*, that acts by inhibiting the mycelial growth of three phytopathogenic fungi: *B. cinerea, F. oxysporum* and *P. piricola*. Moreover, much weaker but not negligible activity was also demonstrated for applanoxidic acid isolated from *Ganoderma annulare* on *Trichophyton mentagrophytes* [56]. It can be concluded that the extracts of both examined species have active substances for suppressing fungal growth; however, considering the variations in activity and potential activity of decreased concentrations in *G. pfeifferi*, a more detailed examination of the extracts at lower concentrations, as well as the purification of individual active components with potential AF action, is necessary.

#### 2.2.3. Antiviral Activity

Animal and plant viruses, as well as bacterial viruses that serve as model organisms for other viruses, are frequently used to assess the AV efficacy of fungal extracts. In this paper, the vB_BbrS_56.1 bacteriophage belonging to the *Siphoviridae* family was used as a model virus for examination of AV activity of four *Ganoderma* species. Both EtOH and H_2_O extracts as well as EtOH extract solution in 5% DMSO were analyzed in the range of concentrations from 0.2 to 200 mg/mL, while the AV activity was presented as the percentage of viable phages for each concentration (Table 4).

All examined *Ganoderma* species (*G. applanatum, G. lucidum, G. pfeifferi* and *G. resinaceum*) exhibited AV activity during incubation for 24 h, at 37 °C in the tested concentration range. Based on the obtained results, the most potent antiviral agents were EtOH extracts dissolved in 5% DMSO, with the emphasis on *G. lucidum* where 73.39 ± 1.35 % inhibition was reached at the lowest tested concentration (0.2 mg/mL). Among EtOH and H_2_O extracts, the highest inhibition potential showed *G. lucidum* and *G. resinaceum*, followed by *G. pfeifferi* which demonstrated moderate activity. On the contrary, *G. applanatum* showed the lowest AV activity in all tested extracts (Table 4).

The high AV activity of *G. lucidum* is in line with a previous study, where triterpenoids (lanosta-7,9(11),24-trien-3-one,15; 26-dihydroxy and ganoderic acid Y) isolated from this species exhibited significant inhibition of enterovirus 71 (EV71) cytotoxicity in human rhabdomyosarcoma cells [28]. Furthermore, AV potency of several ganoderic acids, ganoderol A, ganoderol B, ganodermanondiol, and ganodermanontriol was confirmed in another study [20]. According to the findings of Zhang et al. [28], these triterpenoids prevent AV infection by interacting with the viral particle and preventing the virus from adhering to cells; computer molecular docking revealed that they may bind to the viral capsid protein at a hydrophobic pocket (F site) and prevent EV71 from uncoating. Moreover, in a dose-dependent way, mycelial extracts of *G. resinaceum* decreased the levels of Lake Sinai and honey bee deformed wing viruses [21].

Results obtained in this study showed that if EtOH extracts are not dissolved in a highly non-polar solvent, such as DMSO, its inhibitory potential is decreased. In this case, more non-polar molecules with AV potential are likely to be involved in the activity, which is in accordance with previous studies [35,57]. Terpenoid compounds attributed to AV properties were primarily characterized as active components of *G. lucidum*; however, their presence was also later proven in other species, including *G. colossum, G. applanatum, G. tsugae, G. concinna, G. tropicum* and *G. pfeifferi* [58,59]. Furthemore, the AV activity of MeOH extracts of *G. pfeifferi* against the HSV nie virus (herpes simplex virus I) was demonstrated for isolated compounds of the terpenic and steroid type: aplanoxidic acid, ganoderone, ganoderol, ergosta 7,22-dien-3-ol and lucialdehyde [35]. On the other hand, studies have shown that products rich in phenolic compounds have AV activities against the herpes simplex virus, poliovirus, Coxsackie virus B5 and echovirus 7 [60,61]. For example, caffeic acid, detected in EtOH extracts of all four examined *Ganoderma* species [23], represents one of the polyphenols with AV activity [62]. In summary, the AV potential of mushrooms has been proven not only for extracts, but also for isolated compounds; AV potential is manifested by inhibition of viral enzymes, viral nucleic acid synthesis or adsorption, i.e., inhibition of virus penetration into mammalian cells [63]. Since AV activity of H_2_O extracts is also expressed in *G. pfeifferi*, we can assume that the tested activity is also exhibited by more polar compounds, primarily polysaccharides [64]. Nevertheless, AV activity against HSV virus type 1 and type 2 has been demonstrated for both triterpenes and polysaccharide-protein complexes, while water-soluble and MeOH-soluble components displayed in vitro activity against pathogenic viruses, such as influenza A virus and vesicular stomatitis virus [65,66,67]. Moreover, triterpenoids isolated from *G. lucidum* seem to have anti-HIV activity [67,68], though to create the framework for the use of *G. lucidum* isolates as anti-HIV drugs, extensive studies are still required. There is strong evidence in the literature that triterpenoids, polysaccharides, nucleotides, sterols, steroids, fatty acids and proteins/peptides contained in *G. lucidum* may be helpful for the treatment of COVID-19 despite the lack of clinical data [67]. For example, findings by Al-jumaili et al. [69] show that patients who received *G. lucidum* supplementation also had an increase in lymphocytes; glycosides, which are found in this fungus, also greatly decreased viral reproduction, absorption and penetration to suppress human coronavirus 229E infection in its early stages [67,70].

It can be concluded that the examined extracts of *Ganoderma* species showed the effect of bacteriophage inactivation due to secondary metabolites, such as phenolics and terpenoids. Furthermore, it should be noted that this is the first report of AV activity of *Ganoderma* extracts against vB_BbrS_56.1 bacteriophage (*Siphoviridae* family), which was isolated in 2017 by Petrovic et al. [71]. Further research should be focused on the mechanisms of AV activity, as well as the effect of extracts and/or components on animal viruses.

### 2.3. PCA Analysis

To identify phenolic compounds that are specific to AM activities of each *Ganoderma* extract (EtOH, H_2_O and CHCl_3_), principal component analysis (PCA) was carried out for demonstrated AB, AF and AV activities. To the best of our knowledge, this is the first report of PCA analysis conducted on phenolic compounds and AM activity of selected *Ganoderma* extracts.

**Figure 3:**
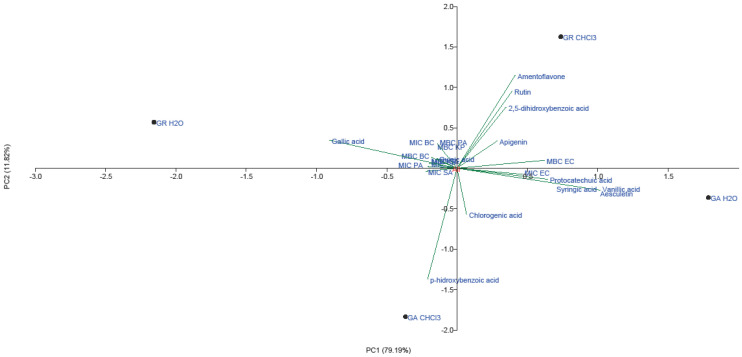
PCA of quantified phenolic compounds and AB activity of CHCl_3_ and H_2_O extracts of *G. applanatum* and *G. resinaceum*. Abbreviations: GA—*G. applanatum*; GR—*G. resinaceum*: MBC—minimal bactericidal concentration (mg/mL); MIC—minimal inhibitory concentration (mg/mL); BC—*B. cereus*; EC—*E. coli*; KP—*K. pneumoniae*; PA—*P. aeruginosa*; SA—*S. aureus*.

Data for microdilution method from Table 1, Table 2 and Table 3 were subjected to PCA for AB activity, with PC1 variance of 79.19% and PC2 accounting for 11.82%. (Figure 3).

Flavonoids, including amentoflavone, rutin and apigenin, had the greatest loading in the positive part of the second principal component, together with 2,5-dihydroxybenzoic acid, gallic acid and quinic acid (Figure 3). Flavonoids and 2,5-dihydroxybenzoic acid grouped in the positive part of both principal components were detected in high amounts in CHCl_3_ extract of *G. resinaceum.* Furthermore, AB activity of *G. resinaceum* CHCl_3_ extract against *E. coli* and *P. aeruginosa* was presented in the positive part of PC1, suggesting that these compounds are probably responsible for obtained activity. In addition, gallic and quinic acid of H_2_O extracts of *G. resinaceum* positively correlated with AB activity against *B. cereus*, *P. aeruginosa* and *K. pneumoniae*. *p*-Hydroxybenzoic acid contributed to the greatest loading in the negative part of both axes and positively correlated with activity of *G. applanatum* CHCl_3_ extract against *S. aureus*. Other phenolic acids and coumarin aesculetin were separated in IV quadrant, together with *G. applanatum* H_2_O extract and MIC against *E. coli*.

**Figure 4:**
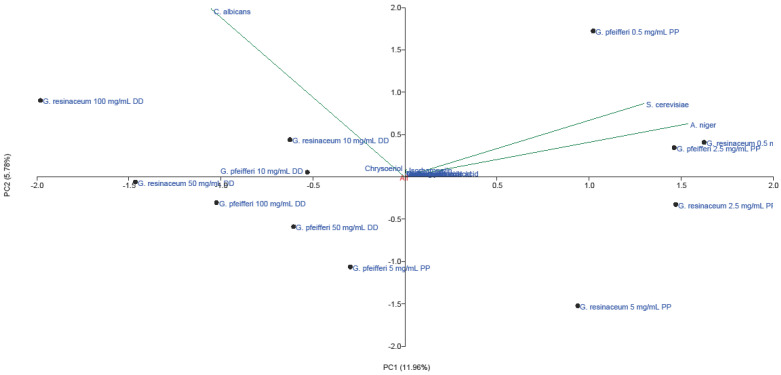
PCA of quantified phenolic compounds and antifungal activity of EtOH extracts of *G. pfeifferi* and *G. resinaceum*. Abbreviations: DD—disk-diffusion method; PP—pour plate method.

The multivariate analysis of the AF activity of EtOH extracts of *G. pfeifferi* and *G. resinaceum* permitted the reduction of the variables to two principal components, with a total variance of 17.74% (Figure 4).

The first axes (PC1) accounted for 11.96% and the second (PC2) for 5.78% of the total variability. Stronger separation was conducted in the horizontal plane of the PC2, with all phenolics and isorhamnetin loading in the II quadrant. This indicates that these compounds positively correlated with high AF activity of tested extracts against *A. niger* and *S. cerevisiae*, using the pour plate method. Low AF effect of these extracts obtained against *C. albicans* (Figure 1 and Figure 2) could be a consequence of low amounts of chrysoeriol, detected only in *G. pfeifferi* [33], since this compound positively correlated with disk-diffusion activity of both *Ganoderma* species against this pathogen.

**Figure 5:**
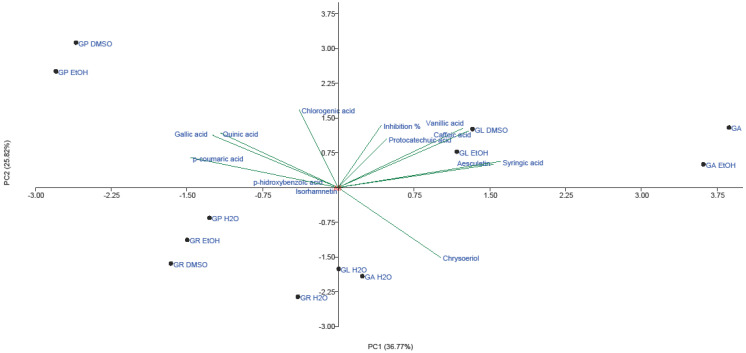
PCA of quantified phenolic compounds and AV activity of both H_2_O and EtOH extracts and EtOH extract solution in 5% DMSO of *G. applanatum*, *G. lucidum*, *G. pfeifferi* and *G. resinaceum*. Abbreviations: GA—*G. applanatum*; GL—*G. lucidum*; GP—*G. pfeifferi*; GR—*G. resinaceum*.

A total variance of 62.59% was observed for AV activity of all four *Ganoderma* species, with PC1 of 36.77% and PC2 of 25.82%. *G. lucidum*, *G. pfeifferi* and *G. applanatum* (EtOH extract and EtOH extract solution in 5% DMSO) were generally separated from *G. resinaceum* extracts and H_2_O extracts (Figure 5).

Hence, we can assume that high AV activity of *G. resinaceum* may be related to some other compounds. On the other hand, the highest inhibition of virions by both *G. lucidum* DMSO solution and EtOH extract is positively correlated with phenolic acids, presented in the II quadrant. Furthermore, the highest loading of gallic, *p*-coumaric, *p*-hydroxybenzoic, quinic and chlorogenic acids is observed in the first quadrant, and thus is related to significant AV activity of *G. pfeifferi*. Moderate AV activity of H_2_O extract *G. applanatum* and *G. lucidum* may be explained by low content of chrysoeriol detected in these species [23] since this flavone positively correlates with AV activity (Figure 5).

## 3. Materials and Methods

### 3.1. Fungal Material

Fruiting bodies of selected *Ganoderma* fungal species were collected in Serbia in September 2010: *G. applanatum* and *G. lucidum* from Morović woods (near Fruška gora Mountain); *G. pfeifferi* from Nature Park Begečka jama in Danube branch; and *G. resinaceum* from Liman (urban location of Novi Sad Town). Determination and identification were carried out under the authority of Dr. Maja Karaman at the Department of Biology and Ecology, University of Novi Sad. The identifications of the four voucher specimens (*G. applanatum*, No. 12-00714; *G. lucidum*, No. 12-00715; *G. pfeifferi*, No. 12-00723; and *G. resinaceum*, No. 12-00722) were made using both macroscopic and microscopic morphological characteristics and particular identification keys [72,73]. The specimens were then deposited at the mycological collection of the BUNS Herbarium (the herbarium of the Department of Biology and Ecology at the University of Novi Sad, Serbia).

### 3.2. Preparation of Extracts

The extracts were prepared according to the procedure of Rašeta et al. [23], whereas the chloroform (CHCl_3_) extracts were prepared in the same way as ethanolic (EtOH) extracts: evaporated EtOH, CHCl_3_ and lyophilized H_2_O extracts were dissolved in distilled H_2_O to a stock concentration of 50 or 100 mg/mL prior to testing for AM activity. To assess antiviral activity, samples were prepared by dissolving evaporated EtOH extracts in 5% DMSO. All samples were stored at +4 °C prior to the analysis.

### 3.3. LC-MS/MS Analysis of Selected Phenolic Compounds

The quantitative analysis of the phenolic composition of CHCl_3_ extracts of *G. applanatum* and *G. resinaceum* was performed using LC-MS/MS technique according to the previously described method [74]. A detailed description is available in Supplementary Data S1.

### 3.4. Antimicrobial Activity

#### 3.4.1. Nutrient Media

The following nutrient media (Torlak, Belgrade, Serbia) were used: nutrient agar (NA) as slanted agar for the cultivation of bacteria that were stored at +4 °C and Müeller Hinton Agar (MHA) medium for determining AB activity. Malt agar—(MA) (Torlak, Belgrade, Serbia) was applied for AF determination.

#### 3.4.2. Antibacterial and Antifungal Activity

##### Bacterial and Fungal Strains

In vitro AB susceptibility of *Ganoderma* H_2_O extracts (100 mg/mL stock conc.) against four strains of Gram-positive bacteria (*Staphylococcus aureus* ^ATCC 255923^, *S. aureus* ^ATCC 6538^, *Enterococcus faecalis* ^ATCC 19433^, *Bacillus cereus* ^ATCC 11778^) and five strains of Gram-negative bacteria (*Escherichia coli* ^ATCC 11775^, *E. coli* ^ATCC 11229^, *Pseudomonas aeruginosa* ^ATCC 3554^, and *Klebsiella aerogenes* ^ATCC 13048^ from standard American Type Culture Collection (ATCC), was assessed using the disk-diffusion, agar-well diffusion and microdilution assay (Table 5). AB susceptibility of the CHCl_3_ extracts of *G. applanatum* and *G. resinaceum* against six bacterial strains (Gram-positive *B. cereus* ^HP^ and *S. aureus* ^ATCC 6538^, and Gram-negative *P. aeruginosa* ^ATCC 3554^, *E. coli* ^ATCC 11229^, *Salmonella enteritidis* ^ATCC 13076^, *Klebsiella pneumoniae* ^HP^) was evaluated using in vitro microdilution assay, while streptomycin was used as the reference compound (Table 5).

EtOH extracts of *G. pfeifferi* and *G. resinaceum* were evaluated for AF activity against laboratory strains *Candida albicans*, *Saccharomyces cerevisiae* and *Aspergillus niger*. Zones of fungal growth inhibition were measured according to the standard microbiological procedures [75,76]. The AF activity was evaluated by applying two methods: disk-diffusion and pour plate.

##### Antibiogram

Antibiograms (Table S2) provided data on bacterial sensitivity to specific antibiotics according to the Kirby–Bauer procedure and the standard CLSI procedure [77]. For the sensitivity test, five antibiotics disks (d = 9 mm) were used, including amikacin, tetracycline, methicillin, kanamycin and ceftriaxone (all at 10 µg/mL). The sensitivity of the following bacterial strains was tested: *B. cereus* ^ATCC 11778^, *E. coli* ^ATCC 11229^, *E. coli* ^ATCC 11775^, *E. faecalis* ^ATCC 19433^, *K. aerogenes* ^ATCC 13048^, *P. aeruginosa* ^ATCC 35554^, *S. aureus* ^ATCC 25923^, and *S. aureus* ^ATCC 6538^ (Table S2). After incubation (37 °C, 24 h), the diameter of the inhibition zone was measured and the sensitivity of each bacterium was evaluated as sensitive (S), intermediate (I) or resistant (R) based on reference tables developed by the manufacturer (Torlak, Belgrade, Serbia) (Table S2).

##### Diffusion Assays

The agar-well diffusion assay was used to determine the AM activity of fungal extracts at a concentration of 100 mg/mL. All solid substrates were prepared in 90 mm Petri plates with approximately 22 mL of MHA, which resulted in a final thickness of 4 mm according to the Kirby-Bauer procedure [78]. After drying the substrate, the agar was drilled with 6 wells with a diameter of 6 mm. After 2 h incubation at room temperature, the plates were treated with 50 µL of each individual fungal extract in three replicates and then incubated at 35 °C for 18–24 h. The substrate was inoculated with 0.1 mL suspension of the tested micro-organism, and dilutions of the extracts were made using dH_2_O as a negative control. The diameters of the inhibitory zones were measured after a 24 h incubation and expressed as a distance (mm), including the initial size of the well (6 mm). When applying the disk-diffusion assay, the procedure was repeated, but instead of “drilling” the medium, “empty” white sterile disks (HiMedia Laboratories, Maharashtra, India; 6 mm) were placed on MHA, on which the extract (25 µL) was applied. The same concentrations (100, 50, 25 and 10 mg/mL) were used for each analyzed extract, in three replicates.

The disk-diffusion method for detection of AF activity was performed in 100 × 15 mm Petri plates. The patches of mycelia of tested fungal strain were applied on Petri plates containing MA. At a distance of 0.5 cm from the edge of the mycelium, white sterile disks (HiMedia Laboratories, Maharashtra, India; 0.6 cm) were placed. A 25 µL measurement of each concentration of *Ganoderma* extracts (10 mg/mL, 50 mg/mL and 100 mg/mL) was applied to the disks. Plates were incubated at 23 °C for 72 h until mycelial growth appeared on control disks. The zone of inhibition around the disks, which manifests AF activity, appears in the shape of a crescent [79].

##### Pour Plate Method

Additionally, another method was used to determine the AF activity of *G. pfeifferi* and *G. resinaceum* species. Three doses of extracts (200 µL, 100 µL, 50 µL) in different concentrations (0.5 mg/mL, 2.5 mg/mL and 5 mg/mL) were used. After sterilization, a medium was cooled to 45 °C and different volumes of extracts were added and mixed rapidly. The mycelia were inoculated centrally, with approximately the same amount placed into each Petri plate, while dH_2_O was used as control. After incubation for 48 h, at 30 °C, the diameter of the mycelial colony was measured, and the inhibition of fungal growth was determined compared to the control.

##### Microdilution Assay

Antibacterial activity was determined by the standard microdilution CLSI procedure [80]. For the assay in a 96-well micro-plate (Spektar, Čačak, Serbia), Müller Hinton broth for bacteria (MHB, Torlak, Beograd, Serbia) was used. Inoculum was prepared from overnight cultures and bacterial suspension was justified with a turbidity of 0.5 McFarland (equivalent to 1.5 × 10^8^ colony-forming units (CFU)/mL)). Double dilutions of *Ganoderma* H_2_O extracts and CHCl_3_ extracts of *G. applanatum* and *G. resinaceum* were prepared in 1% polysorbate (Tween 80) to a final concentration ranging from 6.25 to 200 mg/mL. After 24 h incubation, at 37 °C, 1 mL of 1% TTC (2,3,5-Triphenyl-2H-tetrazolium chloride) solution (HiMedia Laboratories, Maharashtra, India) was dosed into each microwell and the microtiter plates were incubated again for 2 h, at 37 °C. The TTC solution stains the cells pink-red and allows better visualization of the detection of bacterial culture growth in the wells. The minimal inhibitory concentration (MIC) value in the microtiter plates was evaluated according to CLSI procedure [81]. To determine the minimal bactericidal concentration (MBC) value, 100 μL of the mixture from the wells in which there was no visible growth was seeded on Petri plates with MHA and placed in a thermostat for the next 24 h. After incubation, bacterial colonies were visually counted on each Petri plate (CFU)/mL)). The optimal number of colonies is from 30 to 300 on standard Petri plates (90 mm diameter) according to the standard procedure.

#### 3.4.3. Antiviral Activity

DNK virus *Bordetella bronchiseptica* specific bacteriophage—vB_BbrS_LK3 (*Siphoviridae* family) was used for AV analysis. The reference strain of *Bordetella bronchiseptica* ^ATCC 10580^ was used as the original host of phage vB_BbrS_LK3. The phages were multiplied on the appropriate host, precipitated with NaCl (58.4 g/L) and PEG6000 (1:10; *w*/*v*), centrifuged (11,000× *g,* 10 min., +4 °C) and purified by isopycnic ultracentrifugation in a discontinuous CsCl gradient [43]. After dialysis, the phage suspension in SM buffer (50 mM Tris HCl (pH 7.5); 0.1 M NaCl; 8 mM MgSO_4_; 1 mg/mL gelatin) was stored at 4 °C, and the bacterial strain at −70 °C, in a medium with 10% glycerol. For the purposes of the experiment, the bacteria were grown on a liquid Luria–Bertani medium for 24 h, at 37 °C. Bacteriophages, whose abundance in the final volume was 1 × 10^5^ plaque forming unit (PFU)/mL, were mixed with selected *Ganoderma* extracts with work concentration from 0.20 to 200 mg/mL. The control involved the incubation of the bacteriophage with the highest concentration of solvent, without the addition of the extract or the tested substance. The prepared mixtures of phages and extracts were incubated for 30 min, at 37 °C, following the neutralization by 10-step dilution of the treated bacteriophages in the SM buffer. The control was treated identically. After the treatment, the phage titer was determined by the plaque method [82] and the number of viable phages compared to the control was expressed as a percentage.

% inhibition = (1 − N_treatment_/N_control_) × 100

N_treatment_—number of viruses in the treatment (assay);

N_control_—number of viruses in the control (solvent/buffer).

### 3.5. Statistical Analysis

All AM assays were performed in triplicates, and the results are expressed as mean values ± standard deviation (SD). The data that have a normal distribution were subjected to two-way analysis of variance (ANOVA) and multivariate analysis of variance (MANOVA). Tukey’s test was used to determine significant differences (*p* < 0.05) between the extracts. In examination of AV activity, values represented the percentage inhibition (%) of plaque growth by analyzed extracts and, for statistical analysis, Pillai’s Trace statistic test was used together with the Bonferroni correction method; MANOVA was applied as post hoc at *p* < 0.05 and *p* < 0.01. The statistical analysis was performed using the IBM SPSS Statistics software version 22.0 for Windows. Principal component analysis (PCA) provided an overview of the relationships between phenolic compounds and AB, AF and AV activities of the analyzed extracts of selected *Ganoderma* species. PCA analysis was performed using PAST software version 4.03 for Windows, which provided the data’s internal structure and allowed good data dispersion.

## 4. Conclusions

To the best of our knowledge, this is the first report that documented coherence between AM activity and quantified phenolic compounds of selected autochthonous *Ganoderma* species from Serbia and the Balkan region in general. The most effective AB and AF agent among all investigated species was *G. resinaceum*, while *G. lucidum* dominated in AV activity. It is important to mention that *G. resinaceum* H_2_O and CHCl_3_ extracts showed high activity against multi-resistant strains, including *B. cereus, E. coli* and *P. aeruginosa*. Regarding other examined species, *G. pfeifferi* showed moderate AM activity, while *G. applanatum* was recorded as a weak AM agent. High AM activity of *G. resinaceum* was aligned with the detected flavonoids (amentoflavone, apigenin and rutin) and 2,5-dihydroxybenzoic acid, suggesting that phenolic compounds play a key role in tested activities. Possible mechanisms of action could be their ability to complex with extracellular and soluble proteins and with bacterial cell walls, whereas more lipophilic flavonoids might contribute to microbial membrane disruption.

This conclusion was in accordance with PCA analysis, conducted for all three AM activities. However, future research should focus on the AM activity of the identified secondary metabolites, both acting individually and in synergism. The processes behind the AM activity of *Ganoderma* are still largely unclear.

## Figures and Tables

**Table 1:** Determined concentrations of selected phenolic compounds using LC–MS/MS method in examined CHCl_3_ extracts of *G. applanatum* and *G. resinaceum* (µg/g d.w.).

Class	Compound	*G. applanatum*	*G. resinaceum*
Biflavonoid	Amentoflavone	4.13	18.95
Flavone	Apigenin	35.12	47.87
Flavonol	Rutin	26.20	32.18
Hydroxybenzoic acids	*p*-Hydroxybenzoic acid	57.85	<48.85
	2,5-dihydroxybenzoic acid	60.12	79.50
Chlorogenic acid	5-*O*-Caffeoylquinic acid	8.22	7.50

˂Number: detected compound—peak observed, concentration is lower than the LoQ (limit of quantification), but higher than LoD (limit of detection); d.w.—dry weight.

**Table 2:** Antibacterial activity of H_2_O *Ganoderma* extracts using three different antibacterial assays.

	*G. resinaceum*		*G. pfeifferi*		*G. lucidum*		*G. applanatum*	
	Agar-well diffusion assay—inhibition zone (mm)							
*B. cereus* ^ATCC 11778^	13		19		11		13	
*S. aureus* ^ATCC 255923^	11		13		-		16	
*S. aureus* ^ATCC 6538^	7		13		-		-	
*E. faecalis* ^ATCC 19433^	-		-		-		-	
*E. coli* ^ATCC 11775^	-		21		16		-	
*E. coli* ^ATCC 11229^	-		16		7		-	
*P. aeruginosa* ^ATCC 3554^	-		-		-		-	
*K. aerogenes* ^ATCC 13048^	-		-		9		-	
*P. mirabilis* ^ATCC 12453^	-		-		-		-	
	Disk-diffusion assay—inhibition zone (mm)							
*B. cereus* ^ATCC 11778^	10		13		11		-	
*S. aureus* ^ATCC 255923^	11		-		-		21	
*S. aureus* ^ATCC 6538^	-		-		11		9	
*E. faecalis* ^ATCC 19433^	10		-		-		-	
*E. coli* ^ATCC 11775^	7		11		-		-	
*E. coli* ^ATCC 11229^	8		9		-		-	
*P. aeruginosa* ^ATCC 3554^	10		-		-		-	
*K. aerogenes* ^ATCC 13048^	10		10		-		-	
*P. mirabilis* ^ATCC 12453^	-		-		-		-	
	Microdilution assay—MIC * and MBC **							
	MIC (mg/mL)	MBC (mg/mL)	MIC (mg/mL)	MBC (mg/mL)	MIC (mg/mL)	MBC (mg/mL)	MIC (mg/mL)	MBC (mg/mL)
*B. cereus* ^ATCC 11778^	100	100	200	↑ 200	200	↑ 200	100	↑ 100
*S. aureus* ^ATCC 255923^	100	↑ 100	200	↑ 200	-	-	100	↑ 100
*S. aureus* ^ATCC 6538^	100	↑↑ 100	200	↑ 200	-	-	100	↑ 100
*E. faecalis* ^ATCC 19433^	-	-	-	-	-	-	-	-
*E. coli* ^ATCC 11775^	-	-	-	-	-	-	-	-
*E. coli* ^ATCC 11229^	100	↑ 100	100	-	-	-	-	-
*P. aeruginosa* ^ATCC 3554^	-	-	-	-	-	-	-	-
*K. aerogenes* ^ATCC 13048^	-	-	-	-	-	-	-	-
*P. mirabilis* ^ATCC 12453^	100	↑ 100	200	↑ 200	200	↑ 200	-	-

* MIC—minimal inhibitory concentration. ** MBC—minimal bactericidal concentration.

**Table 3:** Antibacterial activity of CHCl_3_ extracts of *G. applanatum* and *G. resinaceum* using microdilution assay.

Bacterial Strain	*G. applanatum*		*G. resinaceum*		Referent Antibiotic- Streptomicin	
	MIC (mg/mL)	MBC (mg/mL)	MIC (mg/mL)	MBC (mg/mL)	MIC (μg/mL)	MBC (μg/mL)
*B. cereus* ^HP^	12.5	25	25	25	8	32
*E. coli* ^ATCC 11229^	12.5	↑↑ 25	12.5	50	4	16
*K. pneumoniae* ^HP^	25	25	25	↑↑ 50	2	8
*P. aeruginosa* ^ATCC 3554^	25	25	6.25	50	16	64
*S. aureus* ^ATCC 6538^	25	25	12.5	25	10.25	1
*S. enteritidis* ^ATCC 13076^	25	25	12.5	25	8	64

MIC—minimal inhibitory concentration; MBC—minimal bactericidal concentration.

**Table 4:** Inhibition of growth of vB_BbrS_LK3 virus by *G. applanatum*, *G. lucidum*, *G. pfeifferi* and *G. resinaceum* extracts.

Work Concentration of Extract (mg/mL)	Inhibition (%)			
	*G. applanatum*	*G. lucidum*	*G. pfeifferi*	*G. resinaceum*
	EtOH extracts			
100	15.73 ± 1.74 ^a^	31.66 ± 1.76 ^a,1^	10.95 ± 0.87 ^b^	33.69 ± 2.55 ^c,1^
50	7.05 ± 0.87 ^a^	33.54 ± 7.31 ^a^	20.22 ± 2.14 ^b^	20.80 ± 3.14 ^c^
20	17.90 ± 0.43 ^a^	24.56 ±2.47 ^a^	25.72 ± 2.30 ^b^	21.96 ± 1.53 ^c^
10	4.87 ± 1.30 ^a^	23.70 ± 0.66 ^a^	n.a.	20.51 ± 3.04 ^c^
2	12.11 ± 0.90 ^a^	37.16 ±1.33 ^a^	28.76 ± 3.39 ^b^	20.22 ± 1.40 ^c^
0.2	16.17 ± 1.57 ^a^	40.93 ± 1.15 ^a^	20.95 ± 2.30 ^b^	20.22 ± 1.33 ^c^
	EtOH extract solution in 5% DMSO			
200	n.a.	n.a.	70.03 ± 3.18 ^a^	n.d.
100	65.83 ± 1.75 ^a^	n.a.	51.12 ± 1.59 ^a^	n.d.
50	70.73 ± 1.70 ^a^	47.62 ± 0.64 ^a^	57.00 ± 4.72 ^a^	n.d.
20	48.46 ± 1.06 ^a^	58.82 ± 3.17 ^a^	17.37 ± 4.15 ^a^	n.d.
10	53.92 ± 3.99 ^a^	55.32 ± 3.26 ^a^	41.60 ± 1.68 ^a^	n.d.
2	57.00 ± 1.35 ^a^	57.00 ± 4.37 ^a^	42.02 ± 1.68 ^a^	n.d.
0.2	n.a.	73.39 ± 1.35 ^a^	n.a.	n.a.
	H_2_O extracts			
100	11.02 ± 1.87 ^a^	3.27 ± 1.15 ^a,1^	22.18 ± 1.31 ^b^	26.26 ± 1.31 ^c,1^
50	20.00 ± 6.41 ^a^	n.d.	18.78 ± 5.93 ^b^	28.30 ± 3.32 ^c^
20	5.72 ± 1.08 ^a^	21.63 ± 0.82 ^a^	19.32 ± 1.03 ^b^	12.79 ± 3.43 ^c^
10	n.d.	29.25 ± 1.55 ^a^	8.16 ± 2.55 ^b^	29.39 ± 3.56 ^c^
2	21.90 ± 0.47 ^a^	27.21 ± 2.62 ^a^	1.77 ± 0.85 ^b^	25.85 ± 3.09 ^c^
0.2	4.35 ± 0.85 ^a^	12.38 ± 4.78 ^a^	10.34 ± 0.62 ^b^	22.99 ± 7.90 ^c^

n.a.—extract is not analyzed; n.d.—not detected. Each value is expressed as mean ± SD. Means with different letters (^a–c^) within columns are significantly different (Pillai’s Trace statistic test, MANOVA, Bonferroni correction method). Significant differences between species were determined by post hoc MANOVA at *p* < 0.05, with first letters (^a–c^) indicating significant differences. Significant differences between examined concentrations of different extracts (EtOH/DMSO/H_2_O) were determined by post hoc MANOVA at *p* < 0.01 and the significant difference was determined in all cases, except in *G. lucidum* and *G. resinaceum* at concentration of 100 mg/mL marked with number (^1^).

**Table 5:** Used assays and antimicrobial activity of different *Ganoderma* species extracts.

Species	Extract Type	Assays	Activity
*G. resinaceum*	H_2_O	Agar-well diffusion	AB
		Disk-diffusion	
		Microdilution	
	CHCl_3_	Microdilution	AB
	EtOH	Disk-diffusion	AF
		Pour plate	
*G. applanatum*	H_2_O	Agar-well diffusion	AB
		Disk-diffusion	
		Microdilution	
	CHCl_3_	Microdilution	AB
*G. pfeifferi*	H_2_O	Agar-well diffusion	AB
		Disk-diffusion	
		Microdilution	
	EtOH	Disk-diffusion	AF
		Pour plate	
*G. lucidum*	H_2_O	Agar-well diffusion	AB
		Disk-diffusion	
		Microdilution	

AB—antibacterial activity; AF—antifungal activity.

## Data Availability

Not applicable.

## Supplementary Materials

The following supporting information can be downloaded at: https://www.mdpi.com/article/10.3390/molecules28073264/s1, Table S1. Summarized LC-MS/MS profile of previusly published selected phenolic compounds in the examined EtOH and H2O extracts of G. applanatum, G. lucidum, G. pfeifferi and G. resinaceuma; Table S2. Antibiogram of analyzed bacterial strains; Supplementary Data S1. LC-MS/MS analysis of selected phenolic compounds.
