# Supplementary material for: Do Ganoderma Species Represent Novel Sources of Phenolic Based Antimicrobial Agents?
Source: Molecules. 2023 Apr 6;28(7):3264. doi: 10.3390/molecules28073264 (PMC10096548; doi:10.3390/molecules28073264)
Supplement: Supplementary file 1 [file molecules-28-03264-s001.zip › molecules-2295806-supplementary.pdf]

Table S1. LC-MS/MS profile of selected phenolic compounds in the examined EtOH and H_2O extracts of *G. applanatum*, *G. lucidum*, *G. pfeifferi* and *G. resinaceum*[a].

Analyzed sample	Amount of detected compound (µg/g d.w.)												Reference**
	p-Hydroxybenzoic acid	Protocatechuic acid	*p*-Coumaric acid	Vanillic acid	Gallic acid	Aesculetin	Caffeic acid	Quinic acid	Syringic acid	Chrysoeriol	Isorhamnetin	Chlorogenic acid	
G. applanatum EtOH	3.82	6.40	0.316	11.40	2.10	4.70	1.90	2.90	9.80	n.d.	n.d.	n.d.	[23]
	4.15	7.50	0.325	12.10	2.21	5.15	2.10	3.05	9.60	<0.10*	<0.40*	<0.30*	[45]
G. applanatum H_2O	<0.30*	1.40	<0.20*	4.50	0.40	0.90	<0.20*	2.50	3.00	n.d.	n.d.	n.d.	[23]
	<0.30*	1.60	<0.20*	4.80	0.50	1.00	<0.20*	3.10	3.20	<0.10*	<0.40*	<0.30*	[45]
G. lucidum EtOH	8.30	22.20	0.50	6.30	0.50	0.90	1.70	6.20	<1.60*	n.d.	n.d.	n.d.	[23]
	9.10	23.20	0.70	7.10	0.40	1.00	2.00	6.90	4.10	<0.10*	<0.40*	<0.30*	[45]
G. lucidum H_2O	1.90	0.90	<0.20*	<4.00*	<0.40*	<0.20*	<0.20*	2.50	<1.60*	n.d.	n.d.	n.d.	[23]
	2.20	1.00	<0.20*	<4.00*	<0.40*	<0.20*	<0.20*	3.00	<1.60*	<0.10*	<0.40*	<0.30*	[45]
G. pfeifferi EtOH	23.00	6.50	1.50	6.50	30.50	n.d.	0.80	8.51	n.d.	n.d.	n.d.	1.26	[25]
	3.30	7.50	1.50	7.60	0.50	<0.20*	1.00	10.90	<1.60*	0.10	0.40	0.30	[45]
G. pfeifferi H_2O	5.10	6.20	1.00	4.50	1.50	n.d.	0.80	6.35	n.d.	n.d.	n.d.	0.80	[25]
	3.00	8.90	0.20	<4.00*	<0.40*	<0.20*	0.50	6.60	<1.60*	<0.10*	<0.40*	0.40	[45]

G. resinaceum	12.20	4.01	0.80	<4.00*	15.85	n.d.	0.40	6.90	n.d.	n.d.	n.d.	<0.30*	[25]
EtOH	2.20	2.00	0.20	<4.00*	<0.40*	<0.20*	0.30	6.90	<1.60*	<0.10*	<0.40*	<0.30*	[45]
G. resinaceum	<0.30*	2.65	0.60	<4.00*	1.20	n.d.	0.25	3.00	n.d.	n.d.	n.d.	<0.30*	[25]
H_2O	<0.30*	0.60	<0.20*	<4.00*	<0.40*	<0.20*	<0.20*	<0.40*	<1.60*	<0.10*	<0.40*	<0.30*	[45]

[a] - Table S1 represents our previously published results [23,25,45]; * < Number: peak was observed for detected compound, but concentration is lower than the LoQ (limit of quantification), and higher than the LoD (limit of detection); EtOH, ethanolic extract; H_2O, water extract; n.d. – compounds are not detected in the analysed extracts.

Table S2. Antibiogram of analyzed bacterial strains.

Bacterial strains	Amikacin	Tetracycline	Methicillin	Kanamycin	Ceftriaxone
B. cereus$^{ATCC\ 11778}$	22(S)	24(I)	0(R)	20(R)	10(R)
E. coli$^{ATCC\ 11775}$	22(S)	25(I)	0(R)	20(R)	29(S)
E. coli$^{ATCC\ 11229}$	23(S)	22(R)	0(R)	22(R)	30(S)
E. faecalis$^{ATCC\ 19433}$	0(R)	30(S)	0(R)	12(R)	16(S)
K. aerogenes$^{ATCC\ 13048}$	21(S)	24(I)	0(R)	19(R)	25(S)
P. aeruginosa$^{ATCC\ 3554}$	21(S)	12(R)	0(R)	0(R)	12(R)
S. aureus$^{ATCC\ 255923}$	25(S)	34(S)	35(S)	25(I)	27(S)
S. aureus$^{ATCC\ 6538}$	21(S)	31(S)	26(S)	28(S)	25(S)

S-sensitive; I-intermediate; R-resistant.

Supplementary Data S1

LC-MS/MS analysis of selected phenolic compounds.

The $CHCl_3$ extracts of *G. applanatum* and *G. pfeifferi* were diluted in a 1:1 premixed solution of water and methanol before analysis to achieve a final concentration of 2 mg/mL. All samples and standards were analyzed using Agilent Technologies (AT) 1200 Series high-performance liquid chromatography coupled with AT 6410A Triple Quad tandem mass spectrometer with electrospray ion source, and controlled by ATMassHunter Workstation software - Data Acquisition (ver. B.03.01). All used compounds were separated using a Zorbax Eclipse XDB-C18 (50 mm 4.6 mm, 1.8 m) quick resolution column maintained at 50 °C by injecting 5 L of the samples/standards into the apparatus. Mobile phase was provided at a flow rate of 1 mL/min in gradient mode (0 min 30% B, 6 min 70% B, 9 min 100% B, 12 min 100% B, re-equilibration time 3 min) and included phase A: 0.05% aqueous formic acid and phase B: methanol. By employing the following ion source parameters-nebulization gas (N2) pressure 50 psi, drying gas (N2) flow 10 L/min and temperature 350 °C, capillary voltage 4 kV, negative polarity—eluted chemicals were discovered by ESI-MS. Data were collected utilizing the improved compound-specific parameters in dynamic MRM mode. Agilent MassHunter Workstation software - Qualitative Analysis was used to determine peak regions for each molecule (ver. B.03.01.). The sample concentrations and calibration curves were calculated and plotted using the OriginLabs Origin Pro (version 9.0) software.
